# Death of Prof. Draper

**Published:** 1883-01-20

**Authors:** 


### *DEATH OF PROFESSOR DRAPER.*

Professor Henry Draper, of New York, died November 20th, 1882. He was connected with the Medical Department of the University of New York.

